# Unconventional effects of long-term storage of microwave-modified chicken egg white lysozyme preparations

**DOI:** 10.1038/s41598-021-89849-2

**Published:** 2021-05-21

**Authors:** Grzegorz Leśnierowski, Tianyu Yang, Renata Cegielska-Radziejewska

**Affiliations:** Department of Food Safety and Quality Management, Faculty of Food Science and Nutrition, Poznan University of Life Sciences, Wojska Polskiego 31, 60-624 Poznan, Poland

**Keywords:** Electrophoresis, Enzymes, Antibiotics, Antifungal agents, Antimicrobial resistance, Antiparasitic agents, Antiviral agents, Cancer

## Abstract

Thermal modification is an effective method that induces significant expansion of the antimicrobial properties and other valuable properties of chicken egg white lysozyme. In our latest research, a new innovative method of enzyme modification was developed, in which microwave radiation was used as an energy source to process liquid lysozyme concentrate (LLC). After modification, high-quality preparations were obtained. However, long-term storage in a concentrated form initiated various processes that caused darkening over time and could also lead to other significant changes to their structure and, consequently, to their functional properties. This necessitated multidirectional research to explain this phenomenon. This paper presents the results of research aimed at assessing the physicochemical changes in the properties of microwave-modified lysozyme in the form of a liquid concentrate after long-term storage under refrigeration conditions. The assessment also considered the conditions under the acidity of the modifying medium and the duration of the microwave modification. The analysis showed that the values of the basic parameters determining the quality and usefulness of the modified enzyme significantly improved during long-term storage of the preparations. The greatest changes were observed in the preparations modified for the longest time and in the most acidic environment (process time 260 s, pH 2.0), the number of oligomers under these conditions increased by 18% after 12 months of holding, and the surface hydrophobicity increased by as much as 31%. In addition, microbiological tests showed that the preparations of microwave-modified lysozyme had an effect on gram-positive bacteria as well as on gram-negative, and this effect was significantly enhanced after 12 months. The results confirm that LLC modification with microwave radiation is a highly efficient method to prepare high-quality and high utility potential lysozyme. Notably, an interesting and important phenomenon was the observation of the unconventional behaviour of the preparations during their long-term storage, which increased their utility potential significantly.

## Introduction

Native lysozyme derived from hen egg white (EC3.2.1.17) is a highly functional monomeric enzyme protein composed of 129 amino acids with highly effective antibacterial activity. This enzyme is often used as a model protein in numerous scientific studies and has aroused increasing attention for practical use due to its multiple valuable properties. The food industry is particularly interested in it, especially since it was recognized by the WHO/FDA as safe for the production and preservation of food^1^. This area of activity primarily uses its antibacterial effect, which is based on the destruction of the β-1,4-glycosidic bond found in the structure of the cell wall of many bacteria. However, this mainly applies to gram-positive bacteria because gram-negative bacteria are resistant to such action because this glycosidic bond is protected by the specific structure of their wall^2^. However, the spectrum of the enzyme's antibacterial activity can be significantly expanded as a result of modifications leading to changes in the protein structure that cause its oligomerization and an increase in surface hydrophobicity^3,4^. The modified lysozyme also has antiviral^5^ and antifungal^6,7^ properties and stimulates the appropriate cells of the body to produce various bioactive compounds to obtain antitumour activity^4,8^. According to the latest reports, by stimulating the formation of interferon-β^4^, the enzyme may be an effective agent against the SARS-Cov-2 virus and thus help fight COVID-19^9,10^. This comprehensive potential of lysozyme, especially the modified form, means that extensive research into new methods of its modification is being carried out to deepen the knowledge about modification techniques and improvements and obtain the most effectively modified form, which will enable the wide practical application of the product. Among the known types of lysozyme modification methods, the best known and most used is the group of thermal methods, which includes one of the newest methods, the so-called microwave method, in which the energy necessary to modify the enzyme comes from microwave radiation^11,12^. It has been shown that a high-quality modified enzyme is obtained under properly selected process conditions, and the method has many advantages including the ease of the process and its control, short modification time, and low cost. In addition, this technique allows for the enzyme to be modified at several times higher concentrations in the solution than in other thermal methods, which makes the process very efficient. Therefore, commercial lysozyme produced as a liquid concentrate (LLC) in which the concentration of lysozyme is approximately 20% can be used for modification. The final product is in the form of a liquid concentrate, which facilitates further practical use. However, it has been observed that some changes occur during storage of the final products, which are manifested in a darkening colour. Therefore, it is important to analyse this phenomenon. The main objective of this study was to evaluate changes in selected physicochemical properties of microwave-modified lysozyme stored in the form of a liquid concentrate for 12 months under refrigeration. The stored preparations were also subjected to tests to assess their antibacterial activity.

## Results and discussion

The research presented in this paper is a continuation of work on a new method that uses microwave radiation as an energy source for thermal modification of lysozyme in an oxidizing environment. The results of previous studies have shown that this kind of energy combined with an oxidation reagent effectively promotes the formation of a significant amount of the oligomeric form of lysozyme by increasing its surface hydrophobicity, which is closely related to the creation of its new properties, including the development of its antimicrobial potential^11,12^. By examining a wide range of different factors influencing the effect of this type of modification, it was shown that the enzyme was best modified in a strongly acid (pH 2, 3, and 4) oxidizing environment (addition of hydrogen peroxide), with a microwave energy power of 270 W. In addition, the modification duration was also important, typically from 180 to 260 s.

To reduce the negative influence of high temperature on the modified enzyme, resorcinol is usually used as a protective substance^13^, so this substance was also used in this case. The modification effect was influenced by the type of starting material used, which is commercially in a dried form but is now increasingly produced in the form of a liquid concentrate (LLC). It has been shown that the modification efficiency using LLC, even with enzyme content reduced from 20 to 10%, can significantly increase compared with that obtained using a dried preparation whose concentration in the modified solution was at most 5%. This is of great importance when the method is used for the commercial production of a modified enzyme because a larger amount of the preparation is obtained, and its liquid concentrated form is more convenient to use^4,13,14^. However, during the storage of these preparations, a unique phenomenon of changing properties was observed. With storage time, the samples became darker, and their hydrolytic activity changed. Therefore, further studies on this modification method included assessment of the changes in the most important physicochemical properties, such as the degree of oligomerization, and changes in the hydrophobic surface and hydrolytic activity after 6 and 12 months of storage at 4–6 °C. The colour of the concentrate was also examined, and microbiological tests were performed. The most important results of these studies have been analysed and are presented in this paper.

**Figure 1:**
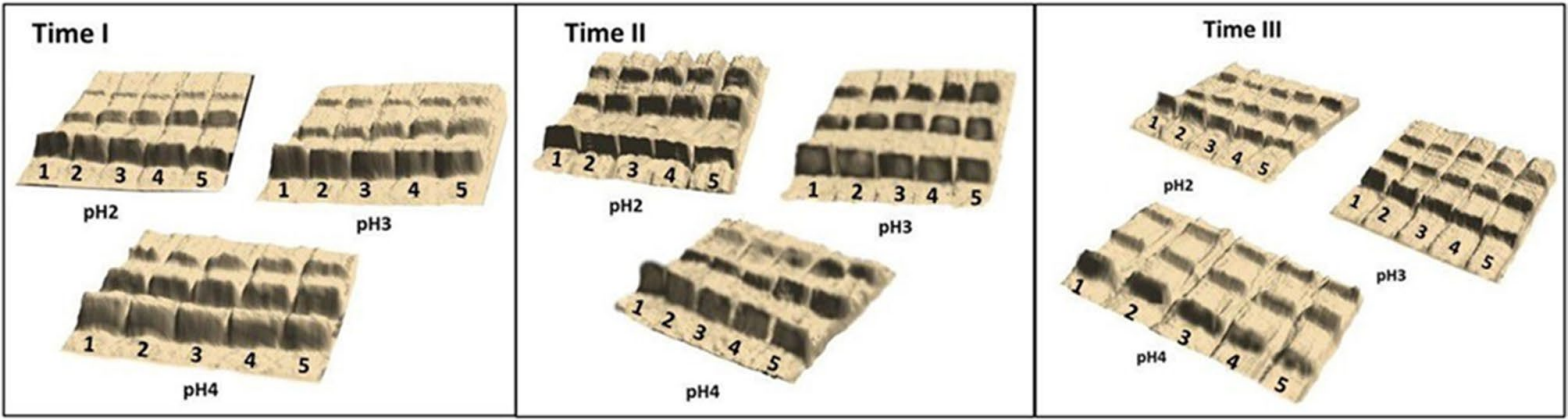
Electrophoretic images (3D) of lysozyme modified by microwave radiation: Time I—directly after modification, Time II—after 6 months of storage, Time III—after 12 months of storage, 1–5—modification time 180, 200, 220, 240, and 260 s respectively.

**Figure 2:**
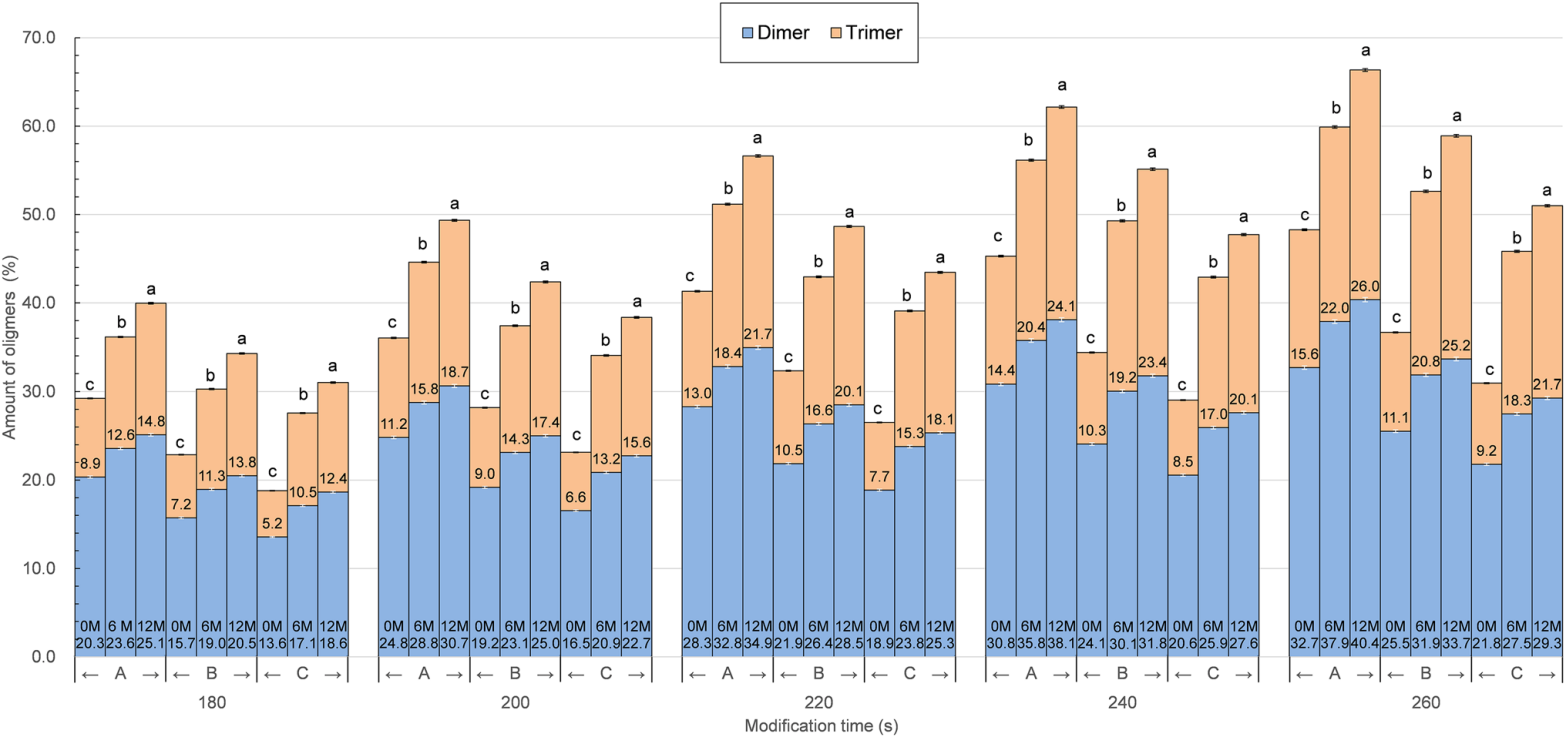
Characterization of the oligomeric properties of an LLC with the modified process, cold storage time, and pH (storage time: 0, 6, and 12 months, time of modification: 180, 200, 220, 240, and 260 s, pH: A-2.0, B-3.0, and C-4.0). (**a**–**c**) Different letters on the bars denote a significant difference in the means at p ≤ 0.05. The top of the bar—trimer. The bottom of the bar—dimer.

The modification of the enzyme was performed according to the procedures outlined in the "Materials and methods" section. The obtained data were collected and subjected to statistical analysis, and the observed correlations and dependencies are presented in the following charts. The influence of the tested factors, i.e., the effects of storage time on the samples modified for 180 to 260 s at pH 2.0, 3.0, and 4.0, on the degree of enzyme oligomerization is graphically illustrated with both 3D images of conducted electrophoresis (Fig. 1) and graphs obtained during data analysis (Fig. 2).

The analysis shows that each of these factors significantly influenced the final modification effect. Oligomerization was most intense in the most acidic environment (pH = 2.0), and the duration of the modification intensified this process; the highest effect was always recorded for the 260-s process. An extremely interesting phenomenon was the further intensive oligomerization of the enzyme during its refrigerated storage. Immediately after modification, under the most favourable conditions (time 260 s, pH 2.0), 48.3% oligomers were obtained, including 32.7% dimer and 15.6% trimer. A similar effect was achieved using other thermal methods^13,14^. However, in the case of the microwave method, the amount of oligomers increased during the storage of samples; after 6 months, it was already 59.9% (37.9% dimer and 22.0% trimer), and after 12 months, it was as high as 66.4% (40.4% dimer and 26.0% trimer). With higher pH values and shorter modification times, these values were lower but the growth rate during storage was similar. Such unconventional behaviour of the modified lysozyme requires additional research aimed at explaining this interesting phenomenon. It will also be important to try to assess the changes that could occur during similar retention of lysozyme modified by other methods but left in a liquid form. The conducted research shows that the key seems to be the form of storage of ready-made preparations, and the modification method only initiates modification processes and affects their effectiveness during the modification process. The research on the mechanism of thermal denaturation of lysozyme shows that protein unfolding resulting from this process causes, among others, the destruction of disulphide bonds, the appearance of free sulfhydryl groups, and the exposure of tryptophan residues to the surface of the molecule, which in turn leads to an increase in the surface hydrophobicity of the enzyme^7,15,16^. Such structural changes in the lysozyme molecule cause its oligomerization with the simultaneous appearance of new antibacterial activity (different from hydrolytic activity) directed mainly against gram-negative bacteria, according to Ibrahim et al.^3,6,17^. On the other hand, the observations of Thomas et al. (1998) show that long-term oxidation of lysozyme in an acidic environment causes its dimerization, and the effect of the process is intensified when a previously thermally modified enzyme is used^18^. In numerous of our laboratory works, we observed similar phenomena^4,11,14,19,20^, which we used in the development of the enzyme modification method presented in this paper. Probably, the combination of all these factors contributed to such an unconventional final effect of storing microwave-modified lysozyme preparations in a liquid oxidizing environment. It can be expected that this effect, resulting in an unexpected increase in the proportion of oligomers and an increase in surface hydrophobicity, should increase the utility value of the preparations because of the greater oligomers and greater hydrophobic surface in the modified enzyme, the greater their antimicrobial activity.

**Figure 3:**
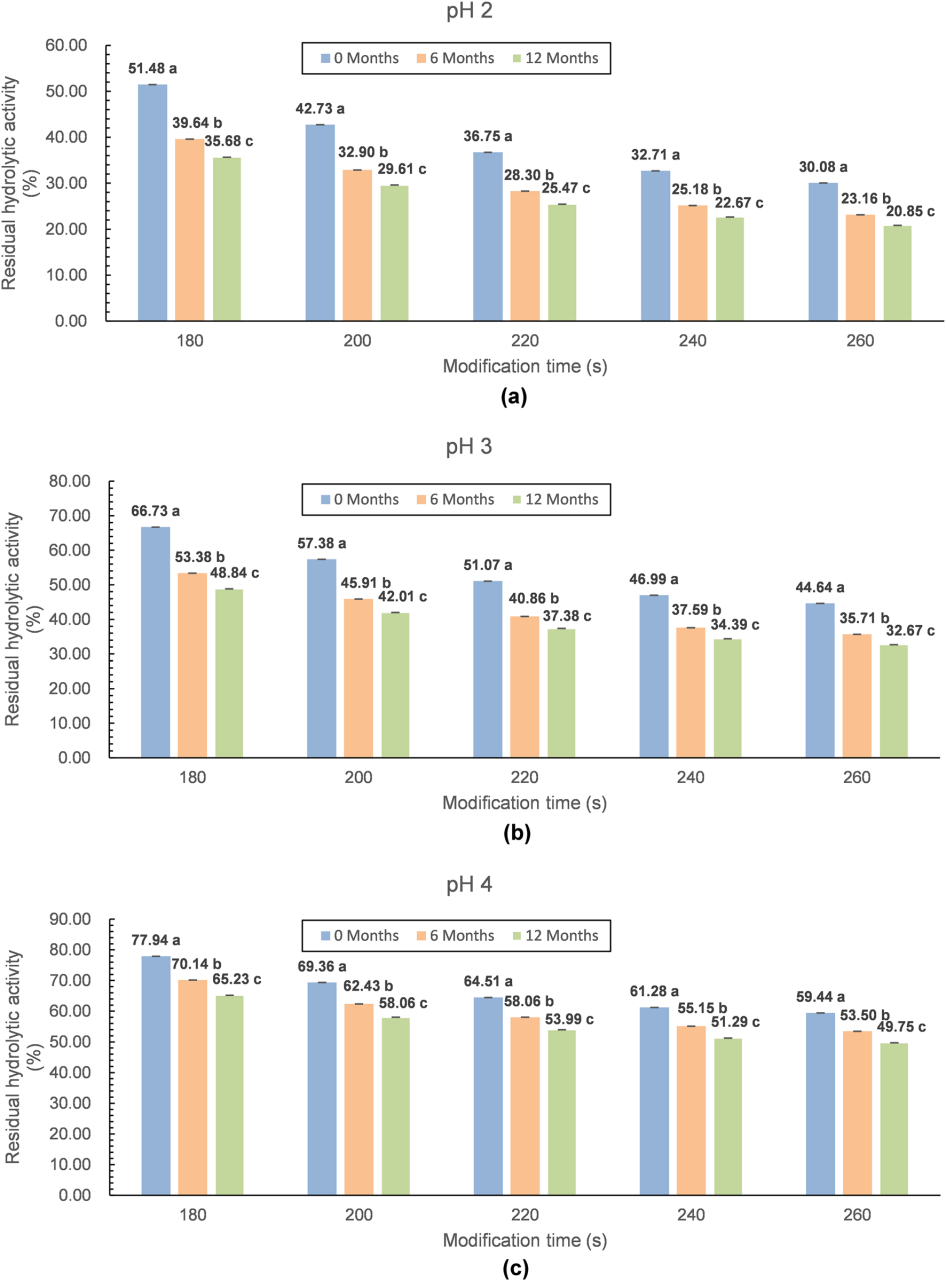
Graphical interpretation of the storage changes of the hydrolytic activity of microwave-modified lysozyme preparations (**a**—pH 2, **b**—pH 3, **c**—pH 4). Storage times: 0, 6, and 12 months. Time of modification: 180, 200, 220, 240, and 260 s. (^a-c^Different letters on the bars denote a significant difference in the means at p ≤ 0.05—separately considered for each modification time).

In contrast, another important physicochemical property, the hydrolytic activity, decreased significantly with increasing storage time. According to Fig. 3, the hydrolytic activity was 77.9% to 30.1% just after modification and 65.2% to 21% that of the natural lysozyme monomer after one year of cold storage. Unlike in the case of oligomerization, the highest decrease in the hydrolytic activity was observed when the pH of the environment was the lowest (pH 2.0), and the modification and storage time were the longest (260 s and 12 months, respectively). Nevertheless, this is not a negative change for the exploration of the potential of lysozyme. Many studies have shown that enzyme activity significantly declines with structural changes in lysozyme when any method is used to stimulate the potential of lysozyme, which is accompanied by the emergence of higher new antimicrobial activity. This newly obtained activity in combination with the remaining catalytic activity creates a completely new antimicrobial potential of the enzyme, so-called total antibacterial activity (TAA), which has a broader spectrum of antimicrobial activity than that of the natural monomer and is also effective against gram-negative bacteria^4,14,21–23^.

**Figure 4:**
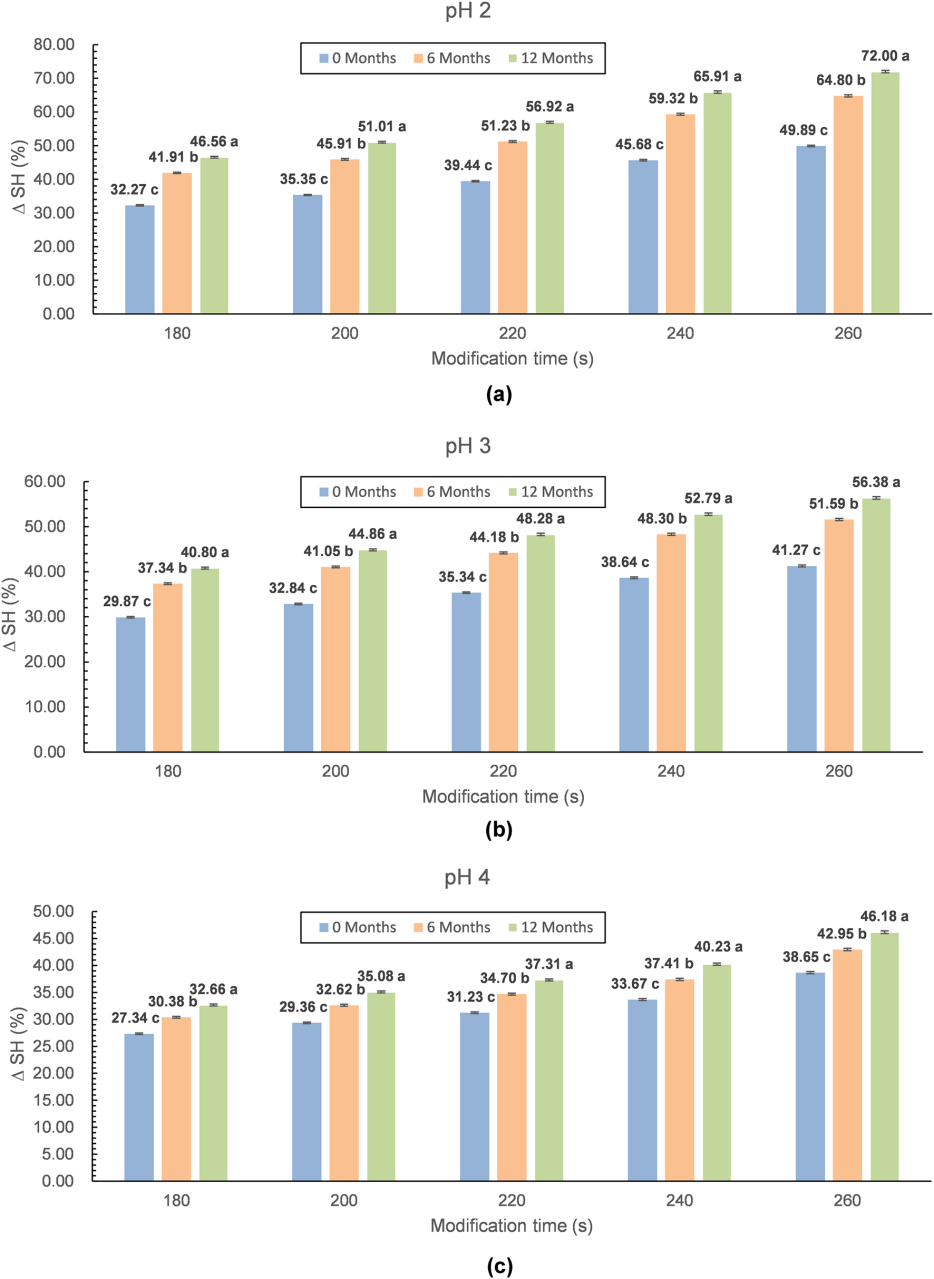
Graphical interpretation of the storage changes of the hydrophobic surface of microwave-modified lysozyme preparations (**a**—pH 2, **b**—pH 3, **c**—pH 4). Storage times: 0, 6, and 12 months. Time of modification: 180, 200, 220, 240, and 260 s. (^a–c^Different letters on the bars denote a significant difference in the means at p ≤ 0.05—separately considered for each modification time).

Although the formation mechanism and principle of new activity are not yet understood completely, one of the factors determining the expansion ability of lysozyme’s new antibacterial spectrum is the oligomer content^3,24,25^. The greater the oligomer content, especially the dimer content, the greater the range of strains that were affected by lysozyme. The change in the hydrophobicity of the lysozyme preparation is generally considered to be one of the most important properties in the new ability of the lysozyme. It was proven that more new antibacterial properties are obtained when the surface hydrophobicity of lysozyme is significantly increased because the modification of lysozyme results in a change in the lysozyme secondary structure and moves the internal location of the hydrophobic group to the surface. This exposure hydrophobicity could increase the binding capacity between lysozyme and the bacterial membrane, increasing the new antibacterial activity^3,26^. Hence, hydrophobicity has an important reference value in judging the changes in the enzyme preparation during storage. By analysing the changes in the hydrophobicity of the lysozyme after modification and during storage (Fig. 4), we noticed the same relationships as those observed for oligomerization; the hydrophobic surface of lysozyme changed obviously during the modification process and long-term storage. With the increase in the degree of enzyme modification (an increase in the amount of oligomers), the surface hydrophobicity also increased, which was consistent with the results of previous studies on the modification of lysozyme using the microwave method^11^. This trend is confirmed by the graphic interpretation of the results presented in Fig. 4, which clearly indicates that the lower the pH was and the longer the modification and storage time were, the more favourable the changes in the hydrophobic surface were. The preparations that were modified for 260 s at pH 2.0 and stored for 12 months under refrigeration were characterized by the highest surface hydrophobicity of over 72%.

**Figure 5:**
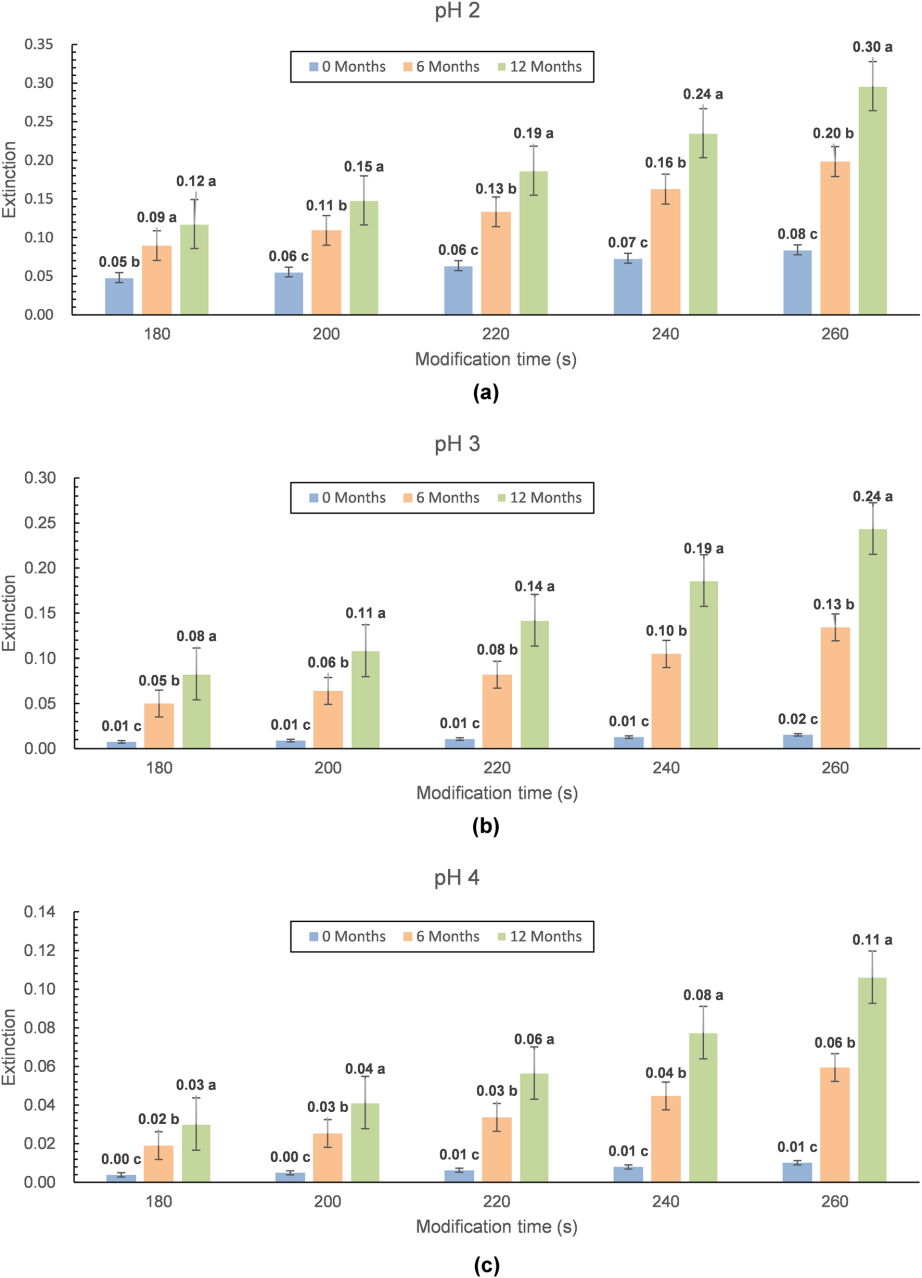
Graphical interpretation of the storage changes in the colour of microwave-modified lysozyme preparations (**a**—pH 2, **b**—pH 3, **c**—pH 4). Storage times: 0, 6, and 12 months. Time of modification: 180, 200, 220, 240, and 260 s. (^a–c^Different letters on the bars denote a significant difference in the means at p ≤ 0.05—separately considered for each modification time).

In the process of storing samples in a cold store, changing their colour was the most easily observed property. Various factors such as storage time, modification time, and pH of the modification process are related to the colour of the lysozyme preparation (Fig. 5). Before modification, the lysozyme solutions were colourless. The modification process caused their darkening, and the intensity of the observed colour changes depended on the conditions of the modification. The cooling storage time has intensified this phenomenon. It was illustrated by changes in spectrophotometrically measured extinctions, the value of which increased from 0.0 for unmodified samples to more than 0.3 for modified preparations stored for 12 months. Statistical analysis showed that, in addition to the storage time, the factors significantly influencing such colour change include a longer time of enzyme modification and a lower pH value of the environment in which it was modified. The cause of such deep changes in the colour of the modified lysozyme is not yet known; therefore, this phenomenon requires additional research. The colour change did not have a significant effect on the tested physicochemical characteristics of the preparations, but it was correlated with them. It is a visible indicator of changes taking place in the modified lysozyme preparations, which can be used to monitor its quality during refrigerated storage.

The analysis of the obtained results shows that the modification of LLC by the microwave method promotes a higher degree of oligomers and increases the surface hydrophobicity in the low-pH modified environment and with longer process time and maintains a significant increasing trend in the degree of oligomers and surface hydrophobicity during a long period of storage (one year). The crucial information resulting from these studies is the fact that the long-term storage of the modified lysozyme improved the desired effects of the original microwave-modified enzyme, increasing its practical utility. Under optimal conditions of a pH of 2.0 and a modification time of 260 s, the proportion of modified lysozyme reached 66.4% (including 40.4% dimer and 26.0% trimer), and the surface hydrophobicity increased to 72%. Although the hydrolytic activity showed a negative change with increasing cold storage time, presumably it was accompanied by the new activity. According to previous studies, lysozyme modifications extend the spectrum of the enzyme's antibacterial action and its TAA is always increased. It has been confirmed that the increase in TAA is characterized by more oligomers and higher hydrophobicity^4,7,27^. Considering the research results, it can be hypothesized that long-term storage of microwave-modified lysozyme under refrigeration conditions (4–6 ℃) may significantly enhance its antibacterial potential and significantly expand the spectrum of its action in addition to very favourable changes in the physicochemical properties. The results of simple microbiological tests carried out on selected lysozyme preparations and bacteria presented in Table 1 verify this hypothesis.

**Table 1:** Microbiological test of lysozyme antibacterial activity against selected gram-positive (+) and gram-negative (−) bacteria.

Sample	Lysozyme concentration inhibiting bacterial growth in suspension	Gram (+) bacteria	Gram (−) bacteria			
		*Micrococcus luteus*	*Escherichia coli*	*Proteus mirabilis*	*Pseudomonas fluorescens*	*Pseudomonas fragi G(−)*
Lysozyme (LLC)	0.5000	+	−	−	−	−
Lysozyme (*directly after modification*)	0.5000	+	+	+	+	+
	0.0625	+	−	−	−	−
Lysozyme (*stored for 12 months*)	0.5000	+	+	+	+	+
	0.0625	+	+	+	+	+

These results clearly show that the modification of LLC with microwave radiation significantly increased the antimicrobial potential of the enzyme. The unmodified lysozyme at a concentration of 0.5% was effective only against gram-positive (+) *Micrococcus luteus *bacteria, whereas it also showed activity at the same concentration against gram-negative (−) *Pseudomonas fluorescens, Pseudomonas fragi, Escherichia coli,* and *Proteus mirabilis* bacteria directly after modification. Even more interesting results were obtained for the same modified preparations when tested after a 12-month storage period. Growth inhibition of all tested bacterial strains was obtained with a 0.0625% enzyme concentration in the suspension. Such positive test results confirmed the need for planning and carrying out extensive microbiological research in this area to reveal the previously unknown, unconventional phenomenon of the increasing antimicrobial potential of the modified enzyme during storage. These results will be disseminated soon.

## Conclusion

The presented results prove that microwave radiation can be an excellent source of energy to produce high-quality modified lysozyme preparations and that the storage of these preparations under refrigeration conditions gives unexpectedly positive effects. The unconventional behaviour of this microwave-modified enzyme has been demonstrated, revealing beneficial changes in its basic physicochemical properties and enhanced antibacterial potential. Due to its unique advantages, this simple and inexpensive method can be an effective tool in the industrial production of these attractive preparations. However, such an unconventional behaviour of microwave-modified lysozyme requires additional research aimed at explaining this interesting phenomenon as well as attempting to assess the changes that could occur during similar storage of lysozyme modified by other methods.

Nevertheless, the results of the presented research reveal new important knowledge about lysozyme, especially its unconventional behaviour during long-term storage after microwave modification and are of unquestionable importance for its practical use in food and other industries, and in medicine, veterinary and pharmacology.

## Materials and methods

### Material

The liquid monomer lysozyme concentrate (LLC) was produced and provided by SACCO Poland company, and the protein content was over 94%. The LLC was distributed as a 20% concentrate high chemical purity preparation and 8800 U/µL hydrolytic activity.

### Microwave modification of LLC

Prior to modification, the lysozyme concentration in LLC was reduced to 10% by diluting the concentrate with distilled water. The pH of the resulting solution was adjusted to 2.0, 3.0, and 4.0 with hydrochloric acid or sodium hydroxide and a protective agent (1.5% benzodiazepine) against enzyme denaturation at high temperature. Hydrogen peroxide was added to each sample as an additional modifier to obtain a concentration of 1.5%. Ten millilitres of each prepared monomer solution (pH 2, 3, and 4) was modified in sealed pressure containers with microwave radiation of 270 W (2.45 GHz) for 180, 200, 240, and 260 s in a microwave integrator (Sharp microwave integrator, model R-978-A). To terminate the modification process completely, the microwave-modified samples were immediately cooled to 0–1 °C. Then, the obtained preparations were stored under refrigeration (4–6 °C). Evaluation of selected physicochemical properties was carried out immediately after the completed modification and after 6 and 12 months of storage.

### Electrophoretic analysis

According to the methods of Laemmli (1970)^28^, the electrophoresis method was performed on 6% thickening polyacrylamide gel and 12.5% split polyacrylamide gel using a Hoefer Scientific Instruments SE-600 (San Francisco, USA) apparatus to assess oligomer formation. After electrophoresis, the gel was fixed for one hour in a solution consisting of 40% water, 50% methanol, and 10% acetic acid. The gel was then stained for 20 h in a 10% acetic acid solution with the addition of 0.25 g Coomassie Brilliant Blue R-250. The gels were scanned and stored as computer files. Next, densitometric analysis was performed using TotalLab Quant computer software by Nonlinear Dynamics Ltd. (United Kingdom, USA).

### Hydrolytic activity

The hydrolytic activity was determined by the spectrophotometric method following the procedure of Leśnierowski et al.^13^. The activity was calculated using the difference in absorbance of the *micrococcus* suspension within 60 s after adding the sample at λ = 450 nm using a VSU2-P Carl Zeiss Jena spectrophotometer (Germany). Each sample was measured 5 times.

The results are presented as the residual hydrolytic activity (RHA), which was calculated as the percentage of the measured hydrolytic activity of modified LLC (HA_L1_) to the hydrolytic activity of natural LLC (HA_L0_)

$${\text{RHA}} = \left( {{\text{HA}}_{{{\text{L1}}}} /{\text{HA}}_{{{\text{L}}0}} } \right)*{1}00\;\left( \% \right)$$

where RHA—is the residual hydrolytic activity, HA_L1_—is the hydrolytic activity of modified LLC, and HA_L0_^—^is the hydrolytic activity of unmodified LLC.

### Surface hydrophobicity of modified lysozyme

The surface hydrophobicity was determined using the ligand agent polyoxyethylene sodium monooleate (Tween 80, SERVA, Germany).

This detergent prevents the Bio-Rad dye from binding to the lysozyme molecule by covering the protein with the hydrophobic site. The level of interference was measured by a spectrophotometer. According to the experimental procedure of Lieske and Konrad (1994)^29^, 0.1% lysozyme solution was diluted with phosphate buffer at pH 6.5 for analysis.

There were four tubes marked a, b, a’, and b’ for each sample.

1. test tube a: 50 µL of lysozyme and 50 µL of distilled water
2. test tube a’: 50 µL of lysozyme and 50 µL of Tween 80 solution
3. test tube b: 100 µL of distilled water
4. test tube b’: 50 µL of distilled water and 50 µL of Tween 80 solution

Each of the test tubes was reacted with 2.5 mL 0.02% Bio-Rad dye solution for 16 min, and then the absorbance was measured at 595 nm wavelength using a Carl Zeiss Jena VSU2-P spectrophotometer.

The surface hydrophobicity (SH) of the modified lysozyme result was calculated according to the formula:

$${\text{SH}} = \frac{{\left[ {a - b} \right] - \left[ {a^{\prime} - b^{\prime}} \right]}}{{\left[ {a - b} \right]}}*100\%$$

The difference in the surface hydrophobicity of the modified SH_Li_ samples and the unmodified LLC sample SH_L0_ was presented as ∆SH.

$$\Delta {\text{SH}} = {\text{SH}}_{{{\text{Li}}}} - {\text{SH}}_{{{\text{L}}0}}$$

where *∆SH* is the change in surface hydrophobicity, SH_L0_ -is the native LLC surface hydrophobicity, and SH_Li_ is the surface hydrophobicity of modified LLC for subsequent samples “i”.

### Colour test

The colour test was based on measurement of the extinction of the obtained preparations solutions and comparison of their size depending on the enzyme modification conditions. Measurements were carried out with the spectrophotometric method using the VSU2-P spectrophotometer by Carl Zeiss Jena (Germany). The test samples were diluted 100 times, and the measurement was carried out at a wavelength λ of 450 nm in quartz cuvettes with an optical path length of 10.0 mm.

### Microbiological test

Both the LLC and microwave-modified enzymes were used in microbiological tests to compare their effectiveness against selected bacterial strains: *Micrococcus luteus* PCM 525 and gram-negative *Escherichia coli* PCM 2793, *Proteus mirabilis* PCM 1361, *Pseudomonas fluorescens* PCM 2123, and *Pseudomonas fragi* PCM 1856. They were provided by the Institute of Immunology and Experimental Therapy of the Polish Academy of Sciences (Wrocław, Poland). From 24-h cultures, bacterial suspensions were prepared in 0.85% NaCl medium (Biomérieux) at a density of 0.5 on the McFarland scale using a Densimat apparatus (Biomérieux). Then, 1 mL bacterial suspension dilution at 10^4^ cfu/ml was added to test tubes containing 4 mL broth and 5 mL prepared solutions of monomer or modified lysozyme. A solution of the required lysozyme concentration was prepared by dissolving lysozyme in sterile water. The test assessment of the antibacterial activity of various forms of lysozyme (unmodified LLC, obtained immediately after modification and modified and stored for 12 months) consisted in determining the threshold concentration of the enzyme at which:

1.Native lysozyme LLC no longer destroyed gram-negative bacteria, and the lysozyme obtained immediately after modification still showed such action.

2.Lysozyme obtained after modification immediately was no longer active against gram-negative bacteria, and the one stored for 12 months was still active.

Samples were incubated at 30 °C or 37 °C for 24 h. After incubation with the classic flooding method, the presence of analysed bacteria was determined on culture media: Mannitol Salt Agar (CM 0085), Pseudomonas Agar Base CM 0559 supplemented with Pseudomonas CFC Selective Agar Supplement (SR 0103), Mc Conkey Agar CM 0115, MRS Agar (De Man, Rogosa, Sharpe), and Mc Conkey Agar CM 0115 (Oxoid). The control comprised samples with no lysozyme solutions.

### Statistical analysis

All results were collected and analysed using SPSS 19.0 software, and the results are presented as the mean values with the standard error. Analysis of variance and post hoc analysis with the Tukey test and Kruskal–Wallis method were used to verify results with heterogeneity of variance. Each test was performed in quintuplicate.
